# Defensive Functioning Moderates the Effects of Nondirective Meditation

**DOI:** 10.3389/fpsyg.2021.629784

**Published:** 2021-01-28

**Authors:** Anne Grete Hersoug, Morten Wærsted, Bjørn Lau

**Affiliations:** ^1^Institute of Clinical Medicine, University of Oslo, Oslo, Norway; ^2^National Institute of Occupational Health, Oslo, Norway; ^3^Department of Psychology, University of Oslo, Oslo, Norway

**Keywords:** defense mechanisms, defensive functioning, stress management, stress reduction, nondirective meditation, worries, mental distress, muscle pain

## Abstract

We have recently found that nondirective meditation facilitates stress reduction. This supplementary study investigated whether defensive functioning would moderate these beneficial effects. We explored the occurrence of defense mechanisms and the impact of defensive functioning on the outcome of companies’ stress management programs regarding worries nervousness, mental distress, sleep problems, and muscle pain. The sample was a population of active, working professionals recruited from Norwegian companies (*n* = 105). The intervention group obtained significant benefits on all outcome measures, but there were no effects in the control group. We analyzed defensive functioning with the self-report questionnaire, Life Style Index, at four time points. The healthy adults who participated had a low level of defense scores at the outset. There was a significant reduction in the level of defenses in both groups over the study period, 6 months. Defensive functioning significantly moderated the change of the outcome measures from baseline to follow-up in the intervention group, but not in the control group.

## Introduction

The concept of defense mechanisms originated within the psychoanalytic tradition, and represents one of the most important contributions made by psychoanalysis to personality theory and psychological adaptation (Freud, 1936, 1959/1894). We use defense mechanisms to cope with demanding emotional experiences. They tend to operate outside of our awareness and may become habitual styles of responding to situations that challenge our self-image and sense of control (Vaillant, 2000). The use of more adaptive defenses is linked to work satisfaction, better mental health, subjective well-being, and better relationships, whereas less adaptive defenses are linked to work problems, difficulty in relationships, and poorer mental health (Larsen et al., 2010).

Increased psychological distress is associated with more use of defenses (Hyphantis et al., 2011). Defense mechanisms also play a role in areas associated with work-related stress. The use of maladaptive defenses predicted emotional exhaustion in a sample of intensive care unit nurses (Regan et al., 2009). The nurses had a predominantly adaptive defense style, and emotional demands itself did not lead to burnout. However, maladaptive defenses appeared to prevent the conscious processing necessary to resolve work-related anxiety, suggesting a mediating role of such defenses.

Endresen et al. (1987) examined occupational groups exposed to both acute and more enduring stress-situations. The main components of the work-related stress experience were role-stress and nonparticipation in decision-making. They found a modulating effect of psychological defenses regarding the experience of psychological stress factors, anxiety, health symptoms, and immunological measures.

Vaernes et al. (1988) investigated psychological defense mechanisms and health among shift-workers in the Norwegian process industry. They found that the combined scores for defenses, perceived health, and work problems explained 25% of the immune measures’ variance. Vaernes et al. (1991) also found associations between stress, psychological factors, health, and immune levels among military aviators.

Unlike many psychological measures, the maturity of defenses is quite independent of social class, education, and IQ (Vaillant, 2000). A sense of social support may be an essential factor linked empirically with both physical health and personality functioning. Defense mechanisms may impact physical health (Olff et al., 1993; Soldz and Vaillant, 1998; Olff, 1999). Maladaptive defenses were positively associated with higher reported levels of life stress, bodily disease, and affective symptoms – and may identify poor copers with high risk for distress (Flannery and Perry, 1990). A sample of back pain patients used more defensive strategies and fewer coping strategies than a sample without such pain. They had more subjective health complaints and less mastery-oriented coping (Eriksen et al., 1997).

Changes in defensive functioning in long-term psychotherapy mostly follow the hierarchy of defense adaptation (Perry and Bond, 2012), suggesting that defenses may be mediating improvement in functioning and symptoms. Addressing defenses during psychotherapy contributes to improved adaptation (Perry and Bond, 2017). Maladaptive defenses were positively and significantly correlated with perceived stress in young and elderly adults (Segal et al., 2007). The results indicated the general stability of adaptive defense mechanisms across the lifespan, and reduced maladaptive defense mechanisms with advancing age.

The present study is a supplement to a study of the effects of a stress reduction technique – nondirective meditation – which yielded significant benefits in the intervention group on all outcome measures: fewer sleep problems, worries mental distress, and musculoskeletal pain (Hersoug et al., 2018). In the control group, no such effects were obtained. The practice of nondirective meditation yielded more effects than only education on stress management. The current study explored defensive functioning in the same non-clinical sample of healthy adults.

### Aims of the Study

The present study investigated defense mechanisms and possible interactions between defensive functioning and the effects of the intervention, nondirective meditation – a stress reduction method. Defense mechanisms were assessed with the Life Style Index (LSI; Plutchik et al., 1979). The study period was 6 months, which was assumed to be too short for significant defensive functioning changes to occur. Therefore, it was expected that the total defense score would be relatively stable (Conte and Plutchik, 1995; Hersoug et al., 2002). Still, some fluctuations over the four repeated measures might be expected, and trends of changes might be observed. We are not aware of any previous studies of this kind, and, therefore, had no specific, empirically based hypotheses regarding possible interactions between defensive functioning and the intervention regarding the outcome measures: worries, mental distress, musculoskeletal pain, and sleep problems. The study was exploratory, without specific hypotheses.

## Materials and Methods

### Participants

The participants were 105 healthy adults who were actively working professionals (Intervention group, *n* = 65; Control group, *n* = 40), who were recruited among employees in five Norwegian companies, comprising insurance, banking, labor and welfare services, and several commercial firms. Table 1 shows baseline data on age, gender distribution, civil status, and level of education. The companies themselves were responsible for recruitment, by inviting employees to learn a nondirective relaxation technique as part of their stress management programs. The companies also recruited participants in the control group (*N* = 47, age 27–64) among employees, mainly from the same departments. In the context of this field study, a randomization procedure for allocating participants to intervention and control was not possible. However, the participants in the control group were informed that they might learn the technique after the 6-month study period.

**Table 1:** Descriptive data at baseline for the intervention and control groups.

	Intervention group	Control group	*T*-test
	*N* = 65	*N* = 40/37^*^	*p*
Age [mean (SD)]	45.6 (8.1)	41.1 (8.4)	0.009
Gender (% females)	72.3	72.5	0.98
Civil status (% married/cohabitating)	63.1	67.5	0.65
Education (% college/university)	86.22	89.2	0.66

* Data on civil status and education missing for three subjects in the control group.

### Study Design and Procedures

#### Stress Management

The intervention and control groups were invited at their worksite to a 2-h seminar on stress, stress responses, and stress management. In each site, the lecture was given collectively to the control and intervention groups. Following the lecture, there was no follow-up for the control group that did not get any stress management teaching. They never participated as a group and never met as a group but filled out questionnaires during the rest of the study. Only the intervention group was pursued by attending an introductory course in Acem Meditation – a nondirective technique (Holen, 2016). Certified teachers guided them through five 2-h sessions over 8 weeks at their workplaces. These meetings were used to meditate (30 min), provide meditation guidance, and discuss meditation experience. Regular, daily practice was recommended, varying from 45 to 30 min per day. After completing the course, the participants carried out the procedure independently without any follow-up during the rest of the study period.

#### A Nondirective Technique – Acem Meditation

Acem Meditation may be described as a nondirective, self-administered type of meditation. A multi-syllable sound with no meaning, neither semantic, mental, nor symbolic, is used as a meditation object. During meditation, this sound is replicated in a soft, effortless manner. A relaxing concentration of consciousness intermittently interrupted by a wandering mind – the attention changes between the meditation object and the material that occurs spontaneously. The meditation sound is “primarily a vehicle to focus internal processes of the mind in a specific way, which can be described either as a state of silent observation, focused attention, heightened awareness of the shifting thoughts and emotions” (Ellingsen and Holen, 2015). The practitioner performs this method with a “free mental attitude,” so that thoughts, memories, emotions, sensations, and moods emerge spontaneously, whether positive or negative. Using such an open-minded approach, what may arise in consciousness is dealt with in an accepting and non-judgmental way. This attitude is characterized by an effortless activity, with no attempt to reduce or alter the spontaneous activity, such as avoiding spontaneous mind wandering. During the course, instructions included how to handle the activities of the mind when changes took place in the inner environment. The training goal was to learn to practice the free mental attitude and retrieve it when lost.

Both groups completed questionnaires at baseline, then after 2, 3, and 6 months. The questionnaires were filled out online. To keep the participants anonymous, a letter was written for each participant, giving them a unique access code. Anonymity was ensured for the research team and others by an engineer administering these procedures. To increase completion rates, general reminders were sent to the participants.

In all four time points, the questionnaires were identical, other than the first one, which also had background data on gender, age, education level, and civil status (single, married, cohabiting, widowed, divorced, or separated). The second completion was made after the end of the 8-week course. There was no follow-up after this time point. The third completion was made 1 month after this, in order to obtain at least three measurements from as many participants as possible, as some attrition was expected at the last follow-up, at the end of the 6 month project period. Besides, all the follow-up questionnaires for the intervention group asked how much the meditation technique was practiced. The selection of instruments for measuring the study results was based empirically on a pilot study (Hersoug et al., 2008). The pilot study – using the same instruments – investigated the effects of a stress reduction program for employees, with significantly better results in the meditation group than the control group – regarding worries, sleep problems, and coping with pressure.

### Instruments

#### Life Style Index

Life Style Index was developed by Plutchik et al. (1979), designed to assess defense mechanisms, assuming that their use is related to specific, affective states and diagnostic concepts. It is a 97 item true-false self-report questionnaire to assess eight defense mechanisms: intellectualization, repression, displacement, compensation, regression, denial, projection, and reaction formation. Investigations of the psychometric properties have supported that it can provide a solid ground for assessing ego defense mechanisms (Endresen, 1991; Hyphantis et al., 2011). The present study used a Norwegian translation by Holen, based on personal communication with Plutchik.

In the statistical analyses, total scores of the 97 defenses were used – i.e., the number of «true» scores were summarized. Higher scores indicated that the subject used more defenses, whereas lower scores indicated fewer «true» defense scores. The use of total LSI scores was deemed appropriate, as previous findings have found that this procedure consistently is the best discriminator among groups (Conte and Plutchik, 1995, p. 197).

#### Eysenck Personality Questionnaire

Eysenck Personality Questionnaire, Neuroticism subscale, EPQ-N, is a self-reported form with 12 items measuring neuroticism, a personal style characterized by worries, nervousness, vulnerability, and tenseness (Eysenck and Eysenck, 1969). This is one of the higher-order factors that EPQ is based on. For this dimension, EPQ-N encompasses the 12 relevant items. The subject fills in “yes” (=1) or “no” (=0) for each item. The EPQ-N yields an overall total score, maximum 12 (high neuroticism).

#### General Health Questionnaire

The 12 item version of the General Health Questionnaire (GHQ-12) is a self-report form (Goldberg et al., 1997) assessing the level of mental distress in the previous 2 weeks. The answers are given as “0” (much less than usual), “1” (less than usual), “2” (same as usual), or “3” (more than usual). The GHQ-12 has an overall total score of up to 36 (high distress). The Norwegian GHQ-12 has demonstrated good psychometric properties (Nerdrum et al., 2006).

#### Bergen Insomnia Scale

Bergen Insomnia Scale (BIS; Pallesen et al., 2008) is a self-report form that contains six items on sleep problems over the last month and on sleep quality and the extent to which sleep quality is satisfactory or unsatisfactory.

#### Musculoskeletal Pain

Self-reported muscle pain intensity for four separate regions of the body was reported for the preceding 4 weeks: neck, shoulders, and upper back; lower back; upper extremities (arms and hands); and lower extremities (hips, legs, knees, and feet). The pain intensity was reported as no pain, mild pain, moderate pain, or severe pain (Hanvold et al., 2010). A sum score of pain in the four body regions was used in the analyses.

### Statistical Analyses

Descriptive statistics were calculated with the LSI scores at baseline and follow-up assessments for the intervention and control groups. The level of defensive functioning was calculated as the total LSI score for the whole sample, the intervention group, and the control group.

Linear mixed model analyses were used to study LSI’s moderating effect on the development of the outcome variables in the intervention group and the control group during follow-up. The analyses were done both unadjusted and with adjustments for age, gender, civil status, and education. Random intercepts were added for company and person, with person nested in the company. Stata SE version 15 was used for linear mixed model analyses and IBM SPSS version 25 for the descriptive analyses.

## Results

### Sociodemographic Data

There were no major differences concerning demographic data between the intervention group and the control group. As shown in Table 1, there were no significant differences between the groups in the distributions of gender, marital status, and education level. However, the intervention group was somewhat older (Table 1).

### Defensive Functioning

At baseline, the intervention group’s total LSI score was 29.95 (SD 8.4), and the control group 27.24 (SD 7.4). In both groups, the total score was significantly lower at the last assessment than at baseline. As shown in Table 2, there were no significant differences in the scores between the two groups.

**Table 2:** Life Style Index (LSI) total score at baseline and three follow-up time points.

	Intervention group			Control group			*T*-test
	*N*	*Mean*	*SD*	*N*	*Mean*	*SD*	*p*
Baseline	64	29.95	8.40	37	27.24	7.43	0.10
Follow-up 2 months	43	25.56	8.14	31	23.06	7.79	0.19
Follow-up 3 months	41	25.41	8.92	24	21.79	8.83	0.12
Follow-up 6 months	30	25.23	10.28	22	20.77	8.62	0.10

### Moderating Impact of Defensive Functioning

In the linear mixed model analysis (Table 3), LSI turned out to be a moderator of the effect of the intervention, nondirective meditation. Significant or near significant effects were found at most of the three follow-up time points. We observed a significant effect for muscle pain at all three time points; for GHQ-12 at 2 and 6 months, and bordering significance at 3 months; for BIS, a significant effect at 2 months, near significance at 3 months; when a preliminary improvement in the control group was also observed, and near significance at 6 months. For EPQ-N, the effect was significant at 6 months, at 3 months near significant, and a nonsignificant effect at 2 months. Adjustments for covariates, age, gender, civil status, and education did not change the result for any of the outcome variables.

**Table 3:** The moderating effect of LSI on the difference between the effects on outcome from baseline to follow-up in the intervention group compared to the control group.

	Sleep (BIS)	EPQ-N	GHQ-12	Muscle pain
**Unadjusted^a^**				
2-month follow-up	−0.13 (*p* = 0.026)	−0.02 (*p* = 0.23)	−0.09 (*p* = 0.017)	−0.04 (*p* = 0.001)
3-month follow-up	−0.11 (*p* = 0.093)	−0.03 (*p* = 0.090)	−0.08 (*p* = 0.063)	−0.03 (*p* = 0.012)
6-month follow-up	−0.11 (*p* = 0.099)	−0.06 (*p* = 0.001)	−0.11 (*p* = 0.013)	−0.03 (*p* = 0.021)
**Adjusted^b^**				
2-month follow-up	−0.13 (*p* = 0.029)	−0.02 (*p* = 0.23)	−0.10 (*p* = 0.014)	−0.04 (*p* < 0.001)
3-month follow-up	−0.10 (*p* = 0.10)	−0.03 (*p* = 0.090)	−0.08 (*p* = 0.059)	−0.03 (*p* = 0.008)
6-month follow-up	−0.11 (*p* = 0.11)	−0.06 (*p* = 0.001)	−0.13 (*p* = 0.010)	−0.03 (*p* = 0.019)

Beta and value of *p* from the linear mixed model analysis.

a Linear mixed model controlling for dependency in data within company and within person (random effects).

b Linear mixed model also adjusting for the covariates age, gender, education level, living alone/cohabitating (fixed effects).

Overall, the analyses indicated moderating impacts of LSI which were significant, or near significant, on the effects of nondirective meditation, on the outcome variables in the intervention group (Table 3).

Beta values were negative at all time points, also when values of *p* were nonsignificant. This may indicate that the estimates of the intervention’s effects for those with higher LSI score were stronger, toward lower outcome scores. The indicator was found at all follow-ups, although not significant at all time points.

## Discussion

### Defensive Functioning

The use of defense mechanisms varies, depending on the current life situation and factors like the level of stress. For those who work under high levels of stress, like the participants in this study, it is likely that use of defenses increases under such circumstances.

There are few studies of defensive functioning, with non-clinical samples of healthy adults. We have found a few, which allow for comparison across studies. In a Norwegian non-clinical sample (*n* = 704), the total defense score was 27.78 (8.5; Endresen, 1991), indicating that these data were in the same range as in the present study. Another Norwegian non-clinical sample had total scores in the same range (Grønningsæter et al., 1991). In comparison, a group of normal elderly persons had a total score of 34.90 (SD 10.48) (Conte and Plutchik, 1995, p. 188); and a group of non-clinical, healthy adolescents 48.88 (SD 29.77; Conte and Plutchik, 1995, p. 193), i.e., the young group used more defenses than the adults in our sample and the sample of elderly persons. Clinical samples had considerably higher defense scores (Conte and Plutchik, 1995, p. 188–193).

### Development of Defensive Functioning

The groups in this study had somewhat different scores at baseline, which is assumed to have impacted the results to some degree, including development of defensive functioning over the course of the project period. The intervention group had a higher initial LSI total score than the control group, and also somewhat higher scores of the outcome variables, which represent stress indicators, suggesting an association between the experience of stress – measured as mental distress, worries, sleep problems, muscle pain, and more use of defenses. With randomization of the participants, such differences between the groups would have been controlled. Furthermore, variability due to multiple data points that were more closely together than desirable, may have played a role in this study. In future studies, multiple measurements over a longer period than 6 months would be preferable.

### Moderating Impact of Defensive Functioning

The analyses yielded systematic indications of a moderating impact of defensive functioning in the group who practiced nondirective meditation – on muscle pain, mental distress, and sleep problems over the study period, but not in the control group. Although there was a significant reduction of defensive functioning in the control group, no moderating impact was observed, and there was no significant improvement on the outcome variables in this group. Thus, the combined impact of the intervention, nondirective meditation, and the change of defensive functioning, was necessary for the significant improvement the intervention group obtained. Recent empirical research supports that change in defenses appears to play a fundamental mediating role in change overall (Perry and Bond, 2017).

### Strengths and Limitations

The inclusion of a control group in a field study of this kind is a strength. The use of multiple data points and several outcome measures is another strength. Another strength of this study was the questionnaires’ computerized management, ensuring anonymity and minimizing the potential impact of evaluator bias. The research team was unable to access the links between the individual participants and the database. Besides, we only used validated and well-known instruments from international research. Diverse instructors taught the courses in different companies in order to reduce personality bias and skill diversities. Participants included active professionals working on their company sites, which fit well with the research questions of the study. They share a stressful situation at work. The statistical analyses were also robust for bias due to subjects leaving during the follow-up by using the linear mixed models.

A conceptual limitation is the use of only a self-report questionnaire, which measures conscious derivatives of unconscious processes. Including a more robust measure of defensive functioning would have increased the strength of the study. Repeated measures of defensive functioning over a longer period than 6 months would have improved the quality of the study. While several measurements with close time points were not a problem regarding the outcome variables, a study of change of defensive functioning improves with measurements that are less close, and over a longer period. Due to the attrition rate after several follow-ups, the study became somewhat underpowered. There were also significantly more women in both groups, and the sample did not represent a balanced gender distribution.

Another limitation that should be mentioned, is the lack of randomization. The context and procedures in this field study did not make it possible to carry out randomization. Because the intervention was carried out as part of the companies’ stress management programs, they recruited both participants to the control and intervention groups. Although the control group participants were offered to learn the meditation technique after the study period, such interests were not required to participate in the control group. Therefore, we cannot ignore that the control and intervention groups may vary in interest and motivation to learn and practice a meditation technique. We are not familiar with factors that differentiate the study participants from non-participants, apart from having time for the course and daily meditation practice. However, in one participating company, both the intervention and control groups had signed up for the intervention. Nonetheless, these analyzes are not shown here; this site’s results were not different from the other companies’ results, indicating that randomization would not have made a big difference in terms of the overall results. Nevertheless, we would encourage others to replicate this study by using an RCT design. Nor can we ignore that participants in the control group could have some positive effect over and above the hypothesized “active” ingredient by non-specific factors such as participating in the project or expecting a later positive impact when obtaining the control condition.

## Conclusion

Defense mechanisms moderated the improvement of mental distress, worries, sleep problems, and muscle pain among working professionals. The defensive functioning may play a role in the process of better stress management. This study is a contribution to more knowledge about defensive functioning, stress reactions, and stress reduction among healthy adults.

## Data Availability Statement

The datasets presented in this article are not readily available because informed consent for sharing data with other researchers was not collected from the participants. Requests to access the datasets should be directed to bjorn.lau@psykologi.uio.no.

## Ethics Statement

The studies involving human participants were reviewed and approved by the Regional Committee for Medical Research Ethics in Eastern and Mid Norway. The patients/participants provided their written informed consent to participate in this study.

## Author Contributions

AH, MW, and BL contributed to the design and manifestation of the study. AH and MW collected the data. MW made the statistical analyses. AH took the main responsibility for writing the manuscript, but all authors contributed and approved the final version of the manuscript. BL took care of the submission and is the corresponding author.

### Conflict of Interest

AH and MW are affiliated to Acem School of Meditation, an international non-profit organization. They serve as unpaid instructors in Acem Meditation in their spare time.

The authors declare that the research was conducted in the absence of any commercial or financial relationships that could be construed as a potential conflict of interest.
